# Airborne spread of severe acute respiratory syndrome coronavirus 2 between rooms in a sealed, mechanically ventilated ward: Evidence from a hospital outbreak investigation

**DOI:** 10.1371/journal.pone.0350608

**Published:** 2026-06-22

**Authors:** Yo Ishigaki, Naohisa Fujita, Tatsuo Kato, Toshiya Ochiai, Haruo Kuroboshi, Akemi Sakane, Norio Asai

**Affiliations:** 1 Research Center for Realizing Sustainable Societies, University of Electro-communications, Tokyo, Japan; 2 Kyoto Okamoto Memorial Hospital, Kyoto, Japan; 3 Department of Infectious Diseases, Kyoto Prefectural University of Medicine, Kyoto, Japan; 4 Air Purification Meister, Shinwa Corporation Co., Ltd., Tokyo, Japan; 5 Japan Air Cleaning Association, Tokyo, Japan; 6 North Medical Center Kyoto Prefectural University of Medicine, Kyoto, Japan; Southwest Jiaotong University, CHINA

## Abstract

Airborne transmission of severe acute respiratory syndrome coronavirus 2 in enclosed, mechanically ventilated hospital wards remains poorly characterized. In February 2025, a coronavirus disease cluster involving 17 individuals occurred across multiple rooms in a sealed Japanese hospital ward. Several infected individuals had no documented close contact with the index patient, raising concerns about ventilation-related airflow-induced inter-room aerosol transmission. A multimodal environmental investigation was conducted via (1) CO_2_ decay experiments to quantify air change rates (ACHs), (2) particulate matter (PM)_2.5_ aerosol dispersion measurements using fog as a surrogate tracer, and (3) computational fluid dynamics (CFD) simulations to visualize airflow and scalar transport. Measurements were taken in the index room (Room A), corridor, and adjacent Rooms B–D under closed- and open-door conditions. Opening the patient room door significantly increased indoor ACHs (3.29/h → 4.01/h, p = 0.030) and allowed CO_2_ tracer gas to escape into the corridor. In the PM_2.5_ dispersion experiment, aerosols released in Room A were detected within the room, corridor, and neighboring rooms, with the highest out-of-room aerosol burden observed at the corridor sensor (area under the curve = 2.6 × 10^5^ μg·s/m^3^). PM_2.5_ and PM_10_ concentrations were strongly correlated (r = 0.9997), revealing intermediate-sized particles capable of longer-range transport. CFD simulations reproduced key qualitative features of the experiments, including tracer accumulation within curtain-enclosed compartments, delayed leakage through the doorway, and downstream transport toward the corridor. Inter-room aerosol transport can occur in sealed, mechanically ventilated wards without natural ventilation or structural openings between rooms. Opening doors improves in-room ventilation and promotes aerosol leakage, revealing a trade-off between the dilution and contamination of shared spaces. Architectural elements such as privacy curtains contribute to airflow stagnation and uneven aerosol removal. Effective infection control strategies must incorporate airflow pathway management and localized filtration to prevent unintended aerosol migration in mechanically ventilated healthcare settings.

## Introduction

The coronavirus disease (COVID-19) pandemic has highlighted airborne transmission as a major route of severe acute respiratory syndrome coronavirus 2 (SARS-CoV-2) transmission. Virus-laden aerosols generated during expiratory activities can remain suspended for extended periods of time and travel beyond conventional distancing thresholds, particularly in enclosed indoor environments. Aerosols <5 µm dominate respiratory emissions and are transported primarily by ventilation-driven airflow rather than gravity [1].

Airborne SARS-CoV-2 RNA has been detected in multiple locations within hospitals, including intensive care units, patient toilets, and protective apparel removal rooms, particularly in locations with insufficient ventilation [2]. Systematic reviews and experimental studies have highlighted the limitations of droplet-centric transmission frameworks and emphasized that ventilation system design, pressure differentials, and airflow pathways critically influence in-hospital transmission dynamics [3–8]. Additional observations in resource-limited facilities show that overlooked ancillary areas, such as waiting rooms, may also become high-risk zones when crowded and poorly ventilated [9]. Surveys among healthcare trainees further indicate persistent gaps in the knowledge of airborne isolation practices [10].

Several outbreak investigations have documented the movement of respiratory aerosols from one room to adjacent spaces within hospitals. For example, Park et al. [11] demonstrated inter-room aerosol movement under open-window natural ventilation conditions; Jung et al. [12] simulated aerosol recirculation within a negative-pressure isolation room originating from the toilet; and a novel CFD analysis explored minimizing virus spread in hospital isolation rooms [13]. Furthermore, de Sousa et al. [14] detected airborne, infectious SARS-CoV-2 in patient rooms and adjoining anterooms.

However, most previous studies were conducted in buildings with operable windows or relied on transient natural ventilation, qualitative visualization of airflow, or limited single-room configurations. Consequently, aerosol behavior in sealed, fully mechanically ventilated hospital wards, which are typical in Japan and many other countries, remains insufficiently researched. Few studies used quantitative environmental tracers to evaluate whether ventilation-driven airflows can facilitate aerosol migration across multiple patient rooms. Airflow-driven transport of airborne contaminants in mechanically ventilated environments is a key consideration in indoor air quality and infection control. Wang et al. [7] reviewed evidence for the airborne transmission of multiple respiratory pathogens, including influenza virus, respiratory syncytial virus, and rhinovirus, and concluded that ventilation design and airflow management are critical engineering controls for reducing indoor transmission risk.

In February 2025, a COVID-19 cluster was identified in the general ward of a mid-sized acute-care hospital in Japan. The ward was fully mechanically ventilated with sealed windows, and several infected individuals had no documented close contact with the index patient, raising suspicion of inter-room airborne transmission.

To investigate this possibility, we conducted an environmental assessment incorporating CO_2_ tracer-gas measurements, particulate matter (PM)_2.5_ aerosol dispersion experiments, and computational fluid dynamics (CFD) simulations. We aimed to determine whether ventilation-driven airflow could account for the multi-room transmission observed during this outbreak.

## Materials and methods

This investigation was conducted in a hospital located in a semi-urban area of Japan with a population of approximately 100,000. The facility employed a fully mechanical ventilation system, and all patient-room windows remained sealed, providing no opportunity for natural ventilation. Each patient room received air from ceiling diffusers, while exhaust air was drawn through washrooms and toilets positioned near the center of the ward. There were no structural openings between the patient rooms, and the central corridor served as a shared passageway for healthcare staff. During the experiments, the temperature was approximately 26°C (range: 23.9–26.8°C), and relative humidity was approximately 43% (range: 38–50%) under routine mechanical ventilation and air conditioning.

**Fig 1:**
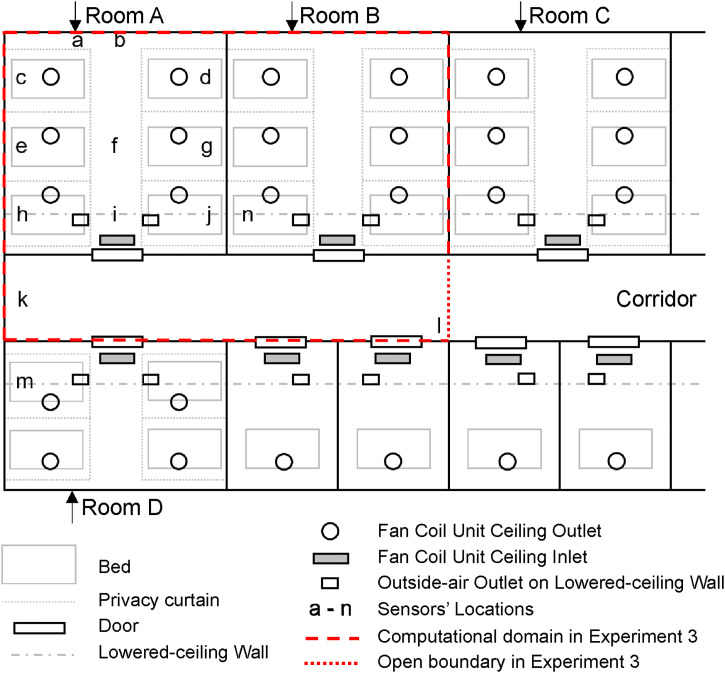
Multi-layered Layout of the COVID-19 Outbreak Ward with Ventilation and Sensor Mapping. This diagram illustrates the physical layout of Rooms A–D, the mechanical ventilation and fan-coil air-conditioning system, the CO_2_ and PM_2.5_ sensor locations (a–n) used in Experiments 1 and 2, and the computational domain analyzed in Experiment 3.

A schematic layout of the affected ward is shown in Fig 1. Four multi-bed rooms (Rooms A–D) situated adjacent to and across each other were connected by a central corridor. The index patient, admitted to Room A (a corner room), developed a fever in February 2025 and subsequently tested positive for SARS-CoV-2. This day was referred to as Day 1. Between Days 1 and 12, additional cases were identified in Rooms B and C, the opposite side of Room D, and among cleaning staff. Several of these individuals had no documented close contact or shared procedures with the index patient.

The Ethics Committee of the University of Electro-Communications, Chofu, Tokyo, Japan, approved this study (Approval No. H23043(3)). All data analyzed in this research were fully anonymized prior to access and analysis; therefore, informed consent was not required. The archived indoor environmental monitoring data used in this study were accessed for research purposes on August 8, 2025. No identifiable personal information was accessible to the authors during or after the data extraction process.

Ultimately, 17 individuals were infected: 12 of the 14 patients staying in Rooms A–D (86%), 4 nurses, and 1 cleaning staff member. The list summarizing the room assignments, occupations, symptom onset, and diagnostic results is presented in Table 1. The spatial distribution and temporal sequence of the cases suggest the possibility of airborne transmission facilitated by inter-room airflow.

**Table 1:** Timeline of COVID-19 cases in a mechanically ventilated ward.

ID	Room	DAY 1	DAY 2	DAY 3	DAY 4	DAY 5	DAY 6	DAY 7	DAY 8	DAY 9	DAY 10	DAY 11	DAY 12
Patient 1 (Index)	A	**AgQ+** DC											
Patient 2	A	**AgQ+** **PCR+** ISO					DC						
Patient 3	A	AgQ-		SX **PCR+**	SX **PCR+**						DC		
Patient 4	A	AgQ-		AgQ-		AgQ-			AgQ-				**AgQ+**
Patient 5	A	AgQ-		AgQ-					DC				
Patient 6	B			SX **AgQ+**					DC				
Patient 7	B			SX **AgQ+**									
Patient 8	B			SX **PCR+**									
Patient 9	B			**AgQ+**	SX **PCR+**								
Patient 10	B							DC	RA **AgQ+**	SX **AgQ+**			
Patient 11	C					SX **Dx+**							
Patient 12	C					AgQ-			DC				
Patient 13	C			AgQ-		AgQ-	SX **PCR+**						
Patient 14	D	DC	**Dx+**										
Nurse 1				SX **Dx+**									
Nurse 2				SX **Dx+**									
Nurse 3						SX **Dx+**							
Nurse 4							SX **Dx+**						
Cleaner 1						SX **Dx+**							

Abbreviations: AgQ + / AgQ− = Quantitative antigen test positive/ negative; PCR+ = RT-PCR positive; Dx+ = Diagnosed with COVID-19; SX = Symptoms consistent with COVID-19; DC = Discharged; RA = Readmitted; ISO = Isolated due to infection control precautions. Rooms A–D are connected to a shared corridor.

The LUMIPULSE G1200 and Lumipulse G SARS-CoV-2 Ag assay kits (Fujirebio Inc., Tokyo, Japan) were used for quantitative antigen detection. According to the manufacturer’s instructions, positivity was determined using a cutoff value of 1.34 pg/mL. Automatic real-time reverse transcription polymerase chain reaction was performed using the GeneXpert System (Cepheid Inc., Sunnyvale, CA, USA) for diagnostic confirmation in selected cases, whereas in others, antigen positivity alone was used as evidence of SARS-CoV-2 infection.

The ventilation system of Room A, where the index patient was assessed, included two ceiling-mounted supply inlets delivering outdoor air (OA) into the room (Fig 1). The total design supply airflow was 252 m^3^/h with a room volume of 85 m^3^, which corresponded to a designed air change rate (ACH) of 3.0/h. Temperature control was provided by a fan coil unit (FCU) with outlets located above each bed, operating in conjunction with ceiling return grilles to circulate air within the room.

Pre-experimental measurements showed that the actual airflow supply from the two inlets was 108.1 m^3^/h and 101.1 m^3^/h, totaling 209.2 m^3^/h (83% of the design specification). Based on the measured airflow, the actual ACH was 2.5/h. This level satisfied the Japanese building requirement for a six-bedroom (180 m^3^/h, equivalent to 30 m^3^/h per person).

Each bed was enclosed within a privacy curtain. The curtain material was impermeable, but there was an approximately 10-cm gap right above the floor and a 30-cm vented mesh section along the upper portion, allowing limited airflow exchange. In routine operations, windows remained closed at all times, doors were kept open, and all privacy curtains were drawn except during patient care activities.

### Experiment 1. CO_2_ decay for ventilation assessment

To evaluate the ventilation performance of the patient rooms, ACH was estimated using the CO_2_ tracer gas decay method, in accordance with Sherman [15] and ASTM E741 [16]. Prior to each experiment, the CO_2_ gas was released into the room for approximately 5 minutes while an electric fan was running to promote air mixing, until the concentration exceeded approximately 5,000 ppm. The fan was then turned off, and CO_2_ decay was measured, under the assumption of a well-mixed condition.

Two types of nondispersive infrared CO_2_ sensors were used: TR-76Ui (measurement range: 0–9,999 ppm; accuracy ±50 ppm or ±5%; T&D Corporation, Nagano, Japan) and the Three Cs Visualization Sensor (measurement range: 400–10,000 ppm; accuracy ±3% + 30 ppm; Asahi Kasei Corp., Tokyo, Japan). Sensors from different manufacturers were employed to avoid depending on a single device type and to enhance the robustness of the measurements. These sensors were installed at 12 predetermined indoor locations (a–l in Fig 1) positioned 60 cm above the floor, and data were recorded at 10-s intervals.

Two experimental conditions were used. In Experiment 1A, the privacy curtains around each bed and the room door were kept closed, representing the usual operating conditions. In Experiment 1B, the curtains remained closed, but the room door was fully open. During both experiments, the FCU and mechanical OA supply systems were operated under routine conditions.

ACH was calculated by fitting the temporal change in the CO_2_ concentration at each sensor location to the exponential decay model shown in Equation (1), where C_0_ represents the CO_2_ concentration (ppm) at the start of ventilation, C_t_ is the concentration after t min, and t is the elapsed time. The background outdoor CO_2_ concentration of 400 ppm was subtracted from C_0_ and C_t_ before analysis.


$$\begin{array}{c} ACH=-\text{6}\text{0}\times \frac{\text{ln}\left(\frac{C_{t}}{C_{\text{0}}}\right)}{t} \end{array}$$ (1)

To compare the ventilation performance between the two-door conditions, a paired t-test was conducted on the ACH values obtained from the indoor sensor locations (a–j).

### Experiment 2. PM_2.5_ tracking for aerosol transport

To evaluate aerosol dispersion from Room A into adjacent indoor spaces, we conducted an aerosol propagation experiment using stage fog as a surrogate particle source. The fog consisted of fine particles readily detectable by PM_2.5_ and was suitable for assessing the spatial aerosol distribution and transport dynamics. The fog was generated using a commercial fog machine (Antari Z-800III, Antari Lighting & Effects, Ltd., Taoyuan, Taiwan) and released from location f near the center of Room A. The fog was used as a qualitative tracer to visualize airflow pathways and relative transport patterns rather than to quantify absolute emission rates.

Particle concentrations were measured using laser-scattering PM_2.5_ sensors (Pocket PM_2.5_ Sensor PRO, Yaguchi Electric Corp., Miyagi, Japan; measurement range: 0–1,000 µg/m^3^; resolution: 1 µg/m^3^) placed at six indoor locations (c, d, e, f, g, i), one corridor location (l), and two locations in neighboring rooms (m, n) (Fig 1). Sensors were positioned 60 cm above the floor, and data were recorded at 1-min intervals.

Two experimental conditions were examined, consistent with those used in Experiment 1. In Experiment 2A, the privacy curtains and room doors were kept closed. In Experiment 2B, the curtains remained closed, while the door was fully open. The measurement duration was 40 min for Experiment 2A and 30 min for Experiment 2B. Fog was emitted only at the beginning of each experiment (70 s in Experiment 2A and 80 s in Experiment 2B), after which the room remained undisturbed. Throughout the experiments, the FCU and mechanical OA ventilation systems were operated under routine conditions.

Following data collection, a zero-offset correction was applied by subtracting the minimum PM_2.5_ recorded by each sensor during the measurement period to estimate the background levels. Time-concentration integrals (area under the curve [AUC]) were calculated for each sensor location.

The PM_2.5_ sensors used in this study also provided simultaneous PM_10_ measurements, allowing the comparison of both particle fractions from a single device. Using these data, Pearson correlation coefficients and coefficients of determination (R²) were calculated to examine the relationship between PM_2.5_ and PM_10_ concentrations during the observation period.

### Experiment 3. CFD analysis

To evaluate the airflow pathways and the potential for inter-room aerosol transport, we performed a three-dimensional CFD analysis using Flowsquare+ (Nora Scientific Inc., Kanagawa, Japan). The computational domain corresponded to the region delineated in Fig 1 (“Computational domain in Experiment 3”) and included Room A and the adjacent section of the corridor. The boundary facing Room B was treated as a solid wall, although Room B was not included. The corridor-side end of the domain was modeled as an open boundary, consistent with the “Open boundary in Experiment 3” (Fig 1).

The simulation used a constant-density isothermal flow model without determining temperature or buoyancy effects, and particle deposition was not included. This simplification was applied to focus on ventilation-driven advection and diffusion as the primary mechanisms of aerosol transport. Although this may affect quantitative estimates (omitting buoyancy may underestimate vertical stratification, and neglecting deposition may lead to slight overestimation of downstream concentrations), these effects were considered secondary to ventilation-driven advection for the purpose of identifying dominant transport pathways. The initial pressure in the computational domain was set to the atmospheric pressure (approximately 1.0 × 10⁵ Pa), and airflow was driven by prescribed velocity boundary conditions at the inlets without imposing additional pressure differences. Air was modeled as a homogeneous fluid with properties representative of room-temperature indoor conditions (density 1.2 kg/m^3^; dynamic viscosity 1.8 × 10^−5^ Pa·s). The physical space (12.7 m × 2.7 m × 8.0 m) was discretized using a uniform Cartesian grid (127 × 27 × 80; grid spacing ~0.1 m). The computation proceeded for a sufficient number of time steps to capture the transient evolution of the flow and scalar fields.

The boundary conditions replicate those of a mechanical ventilation system. A downward inflow of approximately 1.2 m/s was applied at the ceiling outdoor-air supply inlets, and a weaker inflow of approximately 0.1 m/s was applied at the FCU outlets. A suction velocity of approximately 0.013 m/s was applied at the doorway to reproduce the measured airflow toward the corridor. All solid surfaces were treated as no-slip boundaries, and the distal corridor end was modeled as a pressure-release outlet.

Aerosols were represented as a passive scalar that did not undergo gravitational settling, deposition, or coagulation. A normalized scalar concentration (1.0) was supplied from a source region near the room center for the first 80 s, mimicking the fog dispersion experiment (Experiment 2B). After the release stopped, subsequent advection, diffusion, and transport toward the corridor were simulated.

The CFD probe points were placed at the same coordinates as the PM_2.5_ sensors used in Experiment 2B (locations f, i, and l). Then time-series scalar concentrations were extracted from these locations. Flow fields and scalar concentration distributions were visualized along the cross sections indicated in Fig 1 to examine the spatial distribution and transport of the scalar within Room A and toward the corridor.

## Results

### Experiment 1. CO_2_ decay for ventilation assessment

Table 2 summarizes the ACH values calculated from the CO_2_ decay curves obtained under both experimental conditions (Experiments 1A and 1 B). The underlined values represent measurements obtained using the Three Cs Visualization Sensor, while non-underlined values were recorded the TR-76Ui device.

**Table 2:** ACH at each sensor location under two experimental conditions.

Location	Experiment 1A Curtains: Closed Door: Closed	Experiment 1B Curtains: Closed Door: Open
a	3.3/h	5.5/h
b	(3.54)/h	4.6/h
c	2.5/h	2.4/h
d	3.7–3.8/h	4.2/h
e	3.2/h	2.9/h
f	4.1/h	6.5/h
g	3.1–3.4/h	3.1/h
h	2.4–2.5/h	3.1/h
i	4.5/h	4.7/h
j	2.3–2.4/h	3.1/h
k	–	2.6/h
l	–	Not measurable

ACH values were estimated from CO_2_ decay in Experiments 1A (curtains/door closed) and 1B (curtains closed, door open) using an exponential decay model. Underlined: Three Cs Visualization Sensor; others: TR-76Ui. “–”: missing. “Not measurable”: unreliable due to signal issues. (): Mean of two nearby points.

Among the indoor sensor locations (a–j), the mean ACH was 3.29/h in Experiment 1A and 4.01/h in Experiment 1B. The ACH was significantly higher when the room door was open (paired t-test: t = –2.53, p = 0.030; mean difference = 0.72/h).

**Fig 2:**
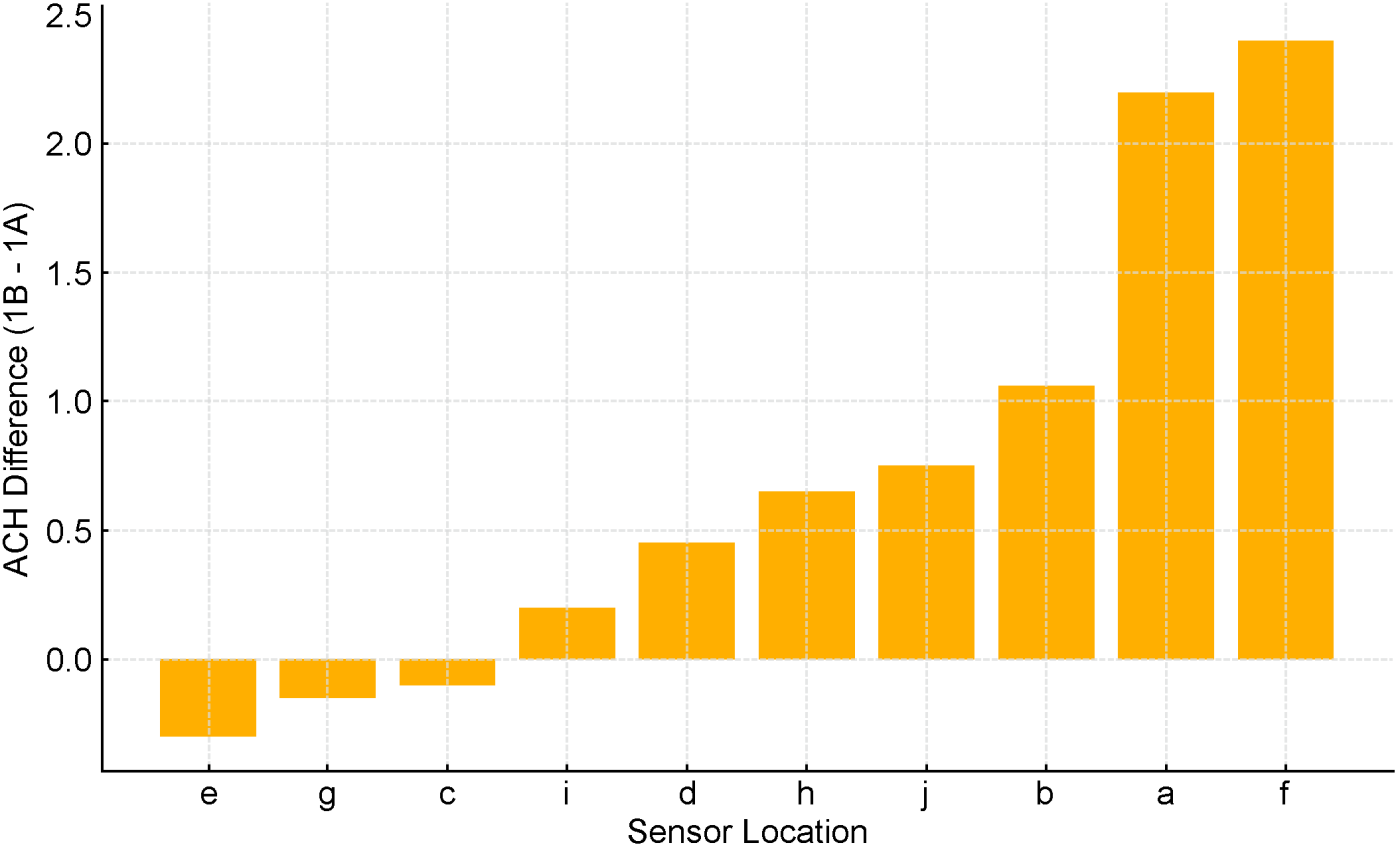
ACH Difference (1B − 1A) at Each Sensor Location (a–j), Sorted in Ascending Order. The bar height represents the difference in ACH between Experiment 1B (curtains closed, door open) and Experiment 1A (curtains and door closed) at each sensor location. Positive values indicate greater ventilation under open-door conditions. Data are shown only for the indoor sensors (locations a–j).

Fig 2 shows the difference in ACH at each location (1B − 1A), arranged in ascending order. Positive values indicate locations where the ventilation performance improved in open-door conditions.

### Experiment 2. PM_2.5_ tracking for aerosol transport

**Fig 3:**
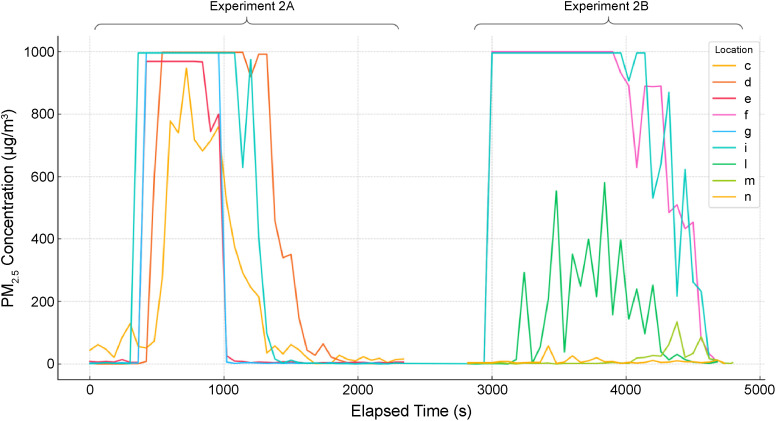
Time-series of Background-corrected PM_2.5_ Concentrations at Each Sensor Location During the Entire Observation Period of Experiments 2A and 2B. Each line represents the time-course of the background-corrected PM_2.5_ concentration at the corresponding sensor location. Offset correction was applied by subtracting the minimum value recorded by each sensor during the measurement period. The figure includes time-series data for both conditions of Experiments 2A (door closed) and 2B (door open), illustrating temporal changes in particle concentration across spatial locations.

**Fig 4:**
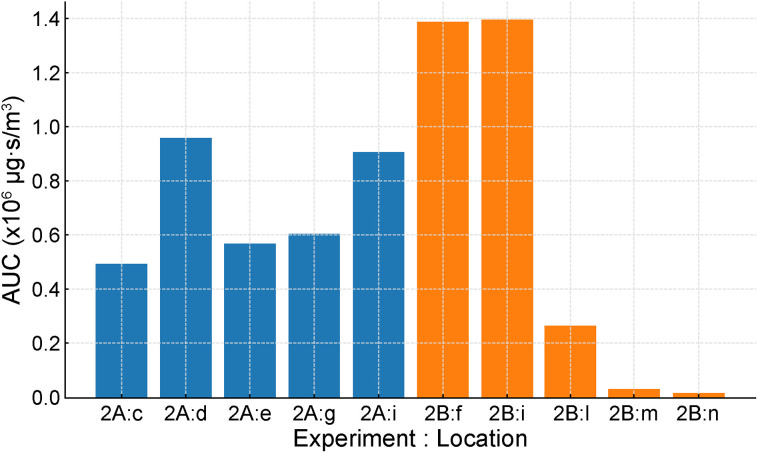
AUC of Background-corrected PM_2.5_ Concentrations at Each Sensor Location in Experiments 2A and 2B. The bar height indicates the AUC of the background-corrected PM_2.5_ concentration at each location. The unit is × 10^6^ μg·s/m^3^. The horizontal axis labels represent the experimental condition and location in the format “2A:c,” “2B:I,”. AUC values were calculated by numerical integration over the observation period based on the offset-corrected concentration.

Fig 3 shows the time-series profiles of background-corrected PM_2.5_ concentrations at each sensor location in Experiments 2A and 2B, and Fig 4 shows the corresponding AUC values.

The simultaneously measured PM_2.5_ and PM_10_ concentrations exhibited an extremely strong correlation (Pearson r = 0.9997, R^2^ = 0.9994). This indicates that most of the detected PM_10_ fraction consisted of particles within the PM_2.5_ size range.

### Experiment 3. CFD analysis

**Fig 5:**
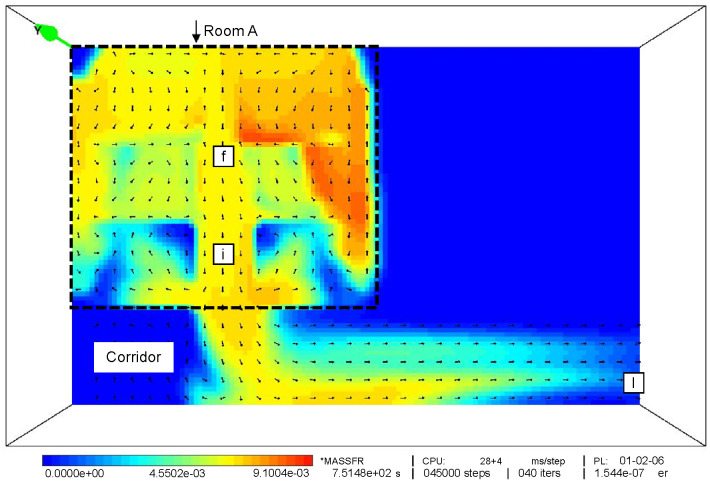
Scalar Concentration Distribution from CFD Simulation of Aerosol Transport.

Fig 5 shows the scalar concentration distribution obtained from the CFD analysis at 750 s (45,000 steps) on a horizontal cross-section at a height of 2.63 m above the floor, near the ceiling. The scalar was dispersed throughout the room and flowed outward through the open doorway toward the corridor. Differences in scalar concentrations were observed within the room in regions partitioned by beds and privacy curtains, and a concentration gradient formed along the downstream transport path toward the corridor. The scalar transported toward the corridor reached the region corresponding to one of the probe locations.

Scalar concentration distribution at 750 s (45,000 steps) on a horizontal cross-section at a height of 2.63 m above the floor, near the ceiling. The warmer colors indicate higher scalar concentrations. Recirculating flow structures developed in Room A, shaped by ceiling-supplied ventilation jets and obstructions formed by beds and privacy curtains. Aerosol-laden air escaped through the doorway and dispersed into the corridor, producing measurable tracer concentrations at the location corresponding to sensor “l.”

**Fig 6:**
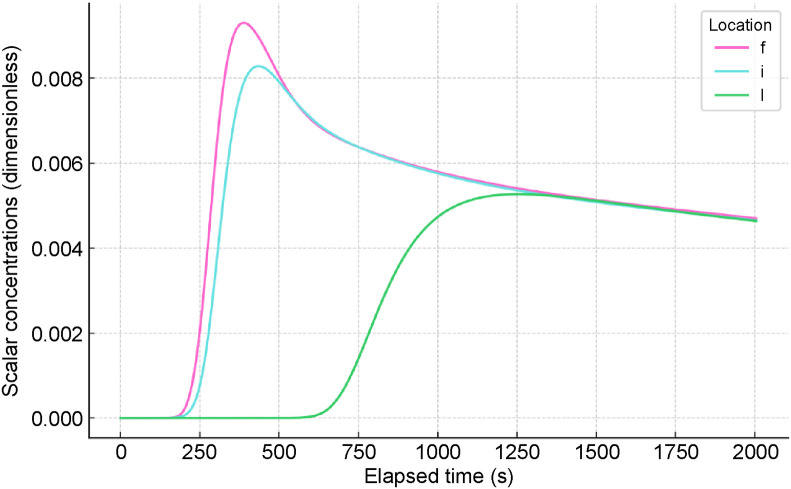
Time-series of Scalar Concentration at Probe Locations f, i, and l in the CFD Simulation. Time-series changes in scalar concentration (mass fraction) at probe points f, i, and **l.** Locations f and i showed an earlier increase in scalar concentration, followed by a later increase at location **l.** The scalar concentrations at all probe points varied over time and gradually decreased after reaching their respective peaks.

Fig 6 presents the time-series scalar concentration (mass fraction) at probe locations f, i, and l. Locations f and i showed an early increase in scalar concentration after the start of the simulation, followed by a later increase at location l. The scalar concentrations at all probe points varied over time and gradually decreased after reaching their respective peaks.

**Fig 7:**
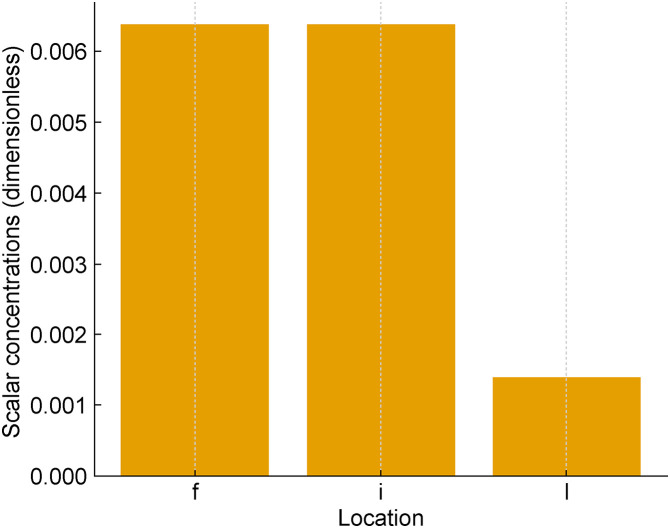
Scalar Concentration Ratios Among Probe Locations f, i, and l at 750 s (45,000 Steps). Scalar concentration (mass fraction) at probe points f, i, and l at 750 s (45,000 steps). The concentrations at f and i were similar in magnitude, whereas the concentration at l was lower. These values show the spatial distribution of scalar concentration across the three probe points at a single time point in the CFD simulation.

Fig 7 shows the scalar concentration ratios at probe locations f, i, and l at approximately 750 s (45,000 steps). The concentrations at locations f and i were similar in magnitude, whereas those at location l were lower. These differences represented the spatial distribution of the scalar concentration among the probe points at a single time point in the CFD simulation.

## Discussion

### Experiment 1. CO_2_ decay for ventilation assessment

Experiment 1 showed that opening the patient-room door increased the ACH, as indicated by the higher decay rates observed under the open-door condition (Fig 2 and Table 2). The increase was not spatially uniform; the locations immediately outside the privacy curtains (a, b, and f) exhibited the largest improvement. This pattern suggests that the curtains created semi-enclosed regions where the airflow was partially restricted under closed-door conditions.

In addition to in-room changes, CO_2_ decay was also detected at the corridor facing location k during the open-door condition, indicating that the tracer gas released in Room A reached the hallway. Fig 2 does not directly show the airflow direction; however, the response at location k provides evidence that the room air leaked into the corridor when the door was open. This finding complements the results from Experiment 2, which demonstrated the transport of PM_2.5_ toward the corridor.

Escombe et al. reported that adding openings, such as doors or windows, increases ventilation efficiency in clinical rooms, consistent with the ACH improvements observed here [17]. Kalliomäki et al. studied the transient airflow associated with door motion rather than sustained open-door conditions, but their work reinforced the broader principle that architectural boundaries strongly influence airflow [18].

The CO_2_ decay profiles showed consistent near-linear trends on a logarithmic scale across multiple sensor locations, indicating near-exponential decay behavior and supporting the practical applicability of assuming well-mixed conditions for ventilation rate estimation. Experiment 1 highlighted that operational practices that enhance in-room ventilation, such as keeping doors open, may concurrently promote outward leakage of indoor air into shared spaces. Thus, both dilution and containment must be considered when managing ventilation in multi-room clinical environments.

### Experiment 2. PM_2.5_ tracking for aerosol transport

Experiment 2A demonstrated that when the door was closed, aerosols remained confined within Room A, with nearly all indoor locations simultaneously reaching the upper detection limit (Fig 3). This pattern indicates that fog particles circulated and accumulated within the room and that the variation in AUC values within room A (up to a two-fold difference; Fig 4) suggests the presence of spatially heterogeneous airflow and localized stagnation zones.

In contrast, Experiment 2B showed a clear sequence of aerosol transport: concentrations first increased at indoor locations f and i and later at corridor location l, the opposite side m, and adjacent room n (Fig 3). The total particle burden was the highest in Room A; however, the AUC values were also elevated at locations outside the source room (Fig 4). Notably, the AUC at the corridor (location l) was the largest among the out-of-room sites, indicating a substantial leakage of fine particles into the shared hallway.

The corridor is commonly used by patients, nurses, and cleaning staff. Long-distance aerosol transport through corridors was previously documented. Ishigaki, et al. [19] reported that CO_2_ tracer gas released from an infected resident’s room in a long-term care facility was transported through the corridor and detected in a distant day room under strong negative-pressure ventilation, illustrating that aerosols can travel several meters through connected indoor spaces.

The particle behavior observed in our experiment is consistent with the “jet rider” mechanism proposed by Hunziker [20]. Jet riders are intermediate-sized droplets (approximately 5–40 µm) that initially behave like aerosols by traveling within a warm, high-momentum turbulent jet (such as cough flow) before settling due to gravity once the airflow decelerates. These particles are poorly captured by conventional ventilation but are efficiently deposited in the nasal mucosa, making them potentially important contributors to airborne transmission. The extremely high correlation between PM_2.5_ and PM_10_ concentrations (r = 0.9997) indicated that particles across the 2.5–10 µm range were present, supporting the interpretation that intermediate-sized particles may have been carried along the airflow pathway extending from Room A into the corridor and adjacent rooms.

These findings show that maintaining a physical distance alone is insufficient to prevent aerosol dissemination in mechanically ventilated wards. Effective infection control strategies must consider airflow pathways, architectural barriers, and particle removal mechanisms to limit cross-room aerosol transport.

### Experiment 3. CFD analysis

The CFD simulation showed aerosol transport patterns that were broadly consistent with the experimental results of Experiment 2B, supporting the validity of the model for interpreting airflow behavior in the ward. Scalar concentrations at probe locations f and i increased earlier than did those at location l (Fig 6), matching the temporal sequence observed in PM_2.5_ measurements (Fig 3). The simulated decay was slower than that in the experiment, which can be attributed to the absence of particle deposition or buoyancy effects. However, the overall progression of the rise, corridor arrival, and gradual decline was qualitatively similar. The relative concentration pattern at approximately 750 s (Fig 7), in which f and i reached similar levels and location l reached approximately one-fifth of those values, was also broadly consistent with the AUC distribution recorded experimentally (Fig 4).

With this level of agreement, the CFD results help to clarify the mechanisms behind the heterogeneous aerosol distribution observed in Experiments 1 and 2. The simulation showed that privacy curtains produced semi-enclosed compartments where the airflow weakened and localized recirculation developed. These stagnant regions correspond to the spatial variability in ACH observed in Experiment 1 and the non-uniform accumulation of PM_2.5_ seen in Experiment 2B. Similar effects were reported in previous studies, where plastic sheets, partitions, and other semi-enclosures were shown to reduce airflow mobility and destabilize local ventilation performance [21–23]. Such compartment-specific stagnation may lead to an uneven infection risk within a room, underscoring the potential value of placing small-high efficiency particulate air-equipped air cleaners inside curtain-enclosed areas to supplement local air exchange and particle removal.

The CFD simulation further demonstrated that scalar escaping from these curtain-defined compartments was transported toward the doorway and entered the corridor. The adjacent rooms were not part of the computational domain; however, the confirmed leakage into the corridor indicated that mechanically ventilated wards may develop indirect airflow continuity between nominally separate rooms mediated by shared corridor spaces. In practice, the corridor functions as a junction of multiple airflow pathways because patient-room doors are frequently open during routine care. This finding aligns with those of prior tracer-gas studies in clinical settings, which showed that contaminants originating from patient rooms can migrate through corridors to distant areas under certain ventilation conditions.

These results highlight the fact that infection control measures cannot rely solely on spatial distance or nominal room-level ventilation rates. Architectural features, such as privacy curtains, and routine operational factors, such as open-door states, substantially influence actual airflow pathways. Strengthening corridor-level containment by installing local exhaust ventilation in shared corridors may help prevent cross-room aerosol migration. Combined with in-room control measures, including localized filtration within semi-enclosed bed spaces, these strategies may reduce the risk posed by unintended airflow connectivity in mechanically ventilated wards.

### Comparison across the three approaches

The three experimental and computational approaches provided mutually reinforcing evidence for ventilation-driven aerosol transport from Room A to the corridor. The temporal sequence of aerosol arrival—first detected at indoor locations f and i, then at corridor location l—was consistently observed in both the PM_2.5_ dispersion experiment (Experiment 2B, Fig 3) and the CFD simulation (Fig 6). The spatially heterogenous ACH values measured under closed-door conditions (Experiment 1A, Table 2) corresponded to the stagnation zones predicted by the CFD model behind privacy curtains, where recirculating airflow and delayed scalar clearance were observed. Furthermore, the distribution of relative scalar concentration at 750 s in the CFD simulation (Fig 7), wherein locations f and i reached similar levels while location l was approximately one-fifth of those values, was broadly consistent with the AUC distribution recorded in Experiment 2B (Fig 4).

Some discrepancies were also identified. The simulated scalar decay was slower than the experimentally observed PM_2.5_ concentration decline, likely because the CFD model did not account for particle deposition on surfaces or gravitational settling. In addition, the computational domain included only Room A and part of the corridor; therefore, the PM_2.5_ signals detected in Rooms B–D during Experiment 2B could not be directly reproduced or quantitatively compared with the simulation results. Despite these limitations, the consistency between the three approaches in identifying the dominant transport pathway—from the source room, through the doorway, and into the corridor—supports the conclusion that mechanically ventilated wards can develop airflow continuity between nominally separate rooms.

### Limitations

In this study, we evaluated airflow and aerosol behavior using multiple complementary approaches; however, several methodological limitations should be acknowledged. First, dynamic factors, such as human movement, door motion, and elevated temperature of exhaled air from febrile patients, were not reproduced or controlled in the experimental protocols or CFD modeling. These factors can substantially influence the transport of CO_2_ and aerosol particles, and their exclusion limits the precision with which real-world dispersions can be quantified. Second, we did not directly measure viable SARS-CoV-2 in the air or on environmental surfaces. Had air or floor sampling been performed, the relationship between aerosol movement and actual infection risk might have been demonstrated empirically. Third, the investigation was conducted in a single ward of an acute-care hospital, and the findings may not be generalizable to facilities with different architectural layouts, ventilation configurations, or operational practices. In addition, the CFD model represented only Room A and a portion of the corridor and did not explicitly incorporate the ventilation ducts or airflow boundary conditions of adjacent rooms. This restricts the interpretation of inter-room transport pathways to the simulated domain. Therefore, interpreting the results requires caution, and further studies across diverse hospital environments are needed.

## Conclusion

In this study, three complementary approaches—CO_2_ decay measurements, PM_2.5_ dispersion experiments, and CFD analysis—were used to characterize airflow and aerosol behavior within a sealed, mechanically ventilated ward. First, the CO_2_ decay analysis demonstrated that opening the patient-room door significantly (p = 0.030) increased ACH while simultaneously allowing room air to leak into the corridor, thereby revealing a trade-off between improved ventilation and increased exposure pathways. Second, PM_2.5_ dispersion experiments showed that aerosols were detected in Room A, the source room, the corridor, and adjacent rooms, providing empirical evidence that airflow continuity through a shared hallway can develop even under mechanical ventilation. Third, the CFD analysis visualized how privacy curtains create semi-enclosed compartments that slow and confine airflow, leading to the local accumulation of aerosols, which subsequently migrate toward the doorway and into the corridor over time. Collectively, these findings demonstrate that spatial distancing alone is insufficient for mitigating airborne transmission risks in mechanically ventilated hospital wards. Infection control strategies must consider airflow pathways shaped by architectural features, incorporate localized filtration within compartmentalized bed spaces, and strengthen the corridor-level exhaust to limit unintended cross-room aerosol transport.
